# A Requirement for Global Transcription Factor Lrp in Licensing Replication of *Vibrio cholerae* Chromosome 2

**DOI:** 10.3389/fmicb.2018.02103

**Published:** 2018-09-10

**Authors:** Peter N. Ciaccia, Revathy Ramachandran, Dhruba K. Chattoraj

**Affiliations:** Laboratory of Biochemistry and Molecular Biology, Center for Cancer Research, National Cancer Institute, National Institutes of Health, Bethesda, MD, United States

**Keywords:** *V. cholerae* Chr2 replication, replication licensing, *crtS*, RctB, Lrp, coordination of replication

## Abstract

The human pathogen, *Vibrio cholerae*, belongs to the 10% of bacteria in which the genome is divided. Each of its two chromosomes, like bacterial chromosomes in general, replicates from a unique origin at fixed times in the cell cycle. Chr1 initiates first, and upon duplication of a site in Chr1, *crtS*, Chr2 replication initiates. Recent *in vivo* experiments demonstrate that *crtS* binds the Chr2-specific initiator RctB and promotes its initiator activity by remodeling it. Compared to the well-defined RctB binding sites in the Chr2 origin, *crtS* is an order of magnitude longer, suggesting that other factors can bind to it. We developed an *in vivo* screen to identify additional *crtS*-binding proteins and identified the global transcription factor, Lrp, as one such protein. Studies *in vivo* and *in vitro* indicate that Lrp binds to *crtS* and facilitates RctB binding to *crtS.* Chr2 replication is severely defective in the absence of Lrp, indicative of a critical role of the transcription factor in licensing Chr2 replication. Since Lrp responds to stresses such as nutrient limitation, its interaction with RctB presumably sensitizes Chr2 replication to the physiological state of the cell.

## Introduction

In bacteria, chromosomes initiate replication at fixed times in the cell cycle that vary depending upon the bacteria and their physiological state. Nearly 10% of bacteria from diverse genera possess divided genomes comprising more than one chromosome (Egan et al., 2005). In such bacteria, timely duplication of all chromosomes prior to cell division is crucial for genome maintenance. *Vibrio cholerae* has emerged as the model organism for studying replication control in multi-chromosome bacteria. It possesses two chromosomes, Chr1, 3 Mb, and Chr2, 1 Mb. Chr1 initiates replication first, and only upon the passage of a Chr1 replication fork across a site, *crtS*, does Chr2 initiate replication (Val et al., 2016). The *crtS* site (Chr2 replication triggering site) is thought to function by interacting with and remodeling the Chr2-specific initiator, RctB (Baek and Chattoraj, 2014). It appears that when duplication of a *crtS* site is prevented within a cell cycle, the site still shows modest activity in licensing Chr2 replication but it is insufficient to do so in a timely fashion (Ramachandran et al., 2018). Duplication of the site as a consequence of a single round of replication increases this activity sufficiently to permit initiation of Chr2 replication in each cell cycle.

The *crtS* site is essential for Chr2 replication in *V. cholerae* (Val et al., 2016). Increasing the copy number of *crtS* increases Chr2 replication in *V. cholerae*, indicating that the activity of the site is limiting for Chr2 replication. The *crtS* site also functions in *Escherichia coli*; the presence of *crtS* in a plasmid increases copy number of plasmids containing the Chr2 origin of replication (p*ori2*) and a source of RctB (Baek and Chattoraj, 2014).

The structure and function of *crtS* are fairly well conserved in the Vibrionaceae family (Kemter et al., 2018). The size of *crtS* (∼153-bp) is rather large for a protein binding site and is much larger than the RctB binding sites in the Chr2 replication origin (12-mers and 39-mers). The region in *crtS* protected by RctB covers only 18 bp (Baek and Chattoraj, 2014). There is plenty of room for other factors to interact with the site. One such factor is RNA polymerase as *crtS* possesses a sigma-70 promoter, called *P_crtS_* here, which remains repressed by unknown factors. The repressed promoter allowed us to screen for host genes responsible for that repression and to determine their influence on *crtS* function.

Here, we show that in addition to RctB, *crtS* binds Lrp, a global transcription factor that responds to nutritional status (Calvo and Matthews, 1994; Cho et al., 2008). The protein is largely responsible for keeping *P_crtS_* repressed and mediating RctB binding to *crtS*. In the absence of Lrp, Chr2 replication is severely defective. The regulation of Chr2 replication by a global regulator of nutritional status may provide a link between chromosomal replication and the physiological state of the cell.

## Materials and Methods

### Bacterial Strains and Growth Conditions

The bacterial strains and plasmids used in this study are listed in **Supplementary Tables S1**,**S2**, respectively. All *E. coli* strains are K12 derivatives and all *V. cholerae* strains are El Tor N16961 derivatives, and were maintained in lysogeny broth (LB) at 37°C and 30°C, respectively, unless otherwise specified. When required, media was supplemented with antibiotics at the following concentrations for *E. coli*: 100 μg/ml ampicillin, 25 μg/ml chloramphenicol, 25 μg/ml kanamycin, 40 μg/ml spectinomycin, and 25 μg/ml zeocin. *V. cholerae* strains were maintained with the same antibiotic concentrations as above except for chloramphenicol, which was used at 5 μg/ml.

### Microscopy

Single colonies grown overnight in LB with the appropriate antibiotics were used to inoculate 1X M63 medium supplemented with 1 mM CaCl_2_, 1 mM MgSO_4_, 0.001% vitamin B1, 0.2% fructose, 0.1% casamino acids, and 100 μM IPTG (to induce GFP-P1ParB). Cultures were grown at 30°C to an OD_600_ of 0.3, added to the center of a glass P35 dish (MatTek corporation, Ashland, MA), and overlaid with 1% agarose prepared with the same medium. Dishes were imaged and analyzed as previously described (Ramachandran et al., 2018).

### Natural Transformation of *V. cholerae* to Replace *crtS* With Δ*3′crtS*, Δ*5′crtS*, or Δ*5*′Δ*3′crtS*

Natural transformations of CVC3058 (HapR^+^ derivative of El Tor N16961 with P1*parS* cloned at +40 kb on Chr2 for visualizing *ori2* as GFP-P1ParB foci) and CVC3061 (CVC3058 with extra *crtS* site cloned 10 kb upstream of native site) were performed as described (Ramachandran et al., 2018). The native copy of *crtS* was replaced with truncated versions using linear DNA amplified from pPC143, pPC144, and pPC145 containing Δ3′*crtS*, Δ5′*crtS*, and Δ5′Δ3′*crtS*, respectively, flanked by 1 kb of homologous DNA present in plasmid pBJH245. pPC143 was assembled from Δ3′*crtS* DNA amplified from pBJH188 with primers PNC47 and PNC56 and from pBJH245 amplified with primers PNC46 and PNC54. Primers used here are described in **Supplementary Table S3**. pPC144 was assembled from Δ5′*crtS* DNA amplified from pBJH188 with primers PNC51 and PNC55 and from pBJH245 amplified with primers PNC50 and PNC53. pPC145 was assembled from Δ5′Δ3′*crtS* DNA amplified from pBJH188 with primers PNC51 and PNC56 and from pBJH245 amplified with primers PNC50 and PNC54. Plasmids were assembled using the HiFi DNA assembly kit (NEB).

### β-Galactosidase Assay

Plasmid Construction: Truncated *crtS* species were transcriptionally fused to *lacZ* in pMLB1109 in order to measure promoter activity. The *crtS* fragments, Δ3′*crtS*, Δ5′*crtS*, and Δ5′Δ3′*crtS* were amplified from pBJH188 using primers PNC15 and PNC17, PNC16 and PNC18, and PNC16 and PNC18, respectively. The fragments were then ligated into pMLB1109 digested with EcoRI and SmaI to produce plasmids pPC066, pPC067, and pPC068, respectively. *E. coli* Δ*lrp* strains was complemented with Lrp using plasmid pPC401. Plasmid pPC401 contained *P_trc_lrp* amplified from pJWD-2 using primers PNC139 and PNC140 in a pACYC177 backbone that was amplified using primers PNC141 and PNC142.

Assay Protocol: β-galactosidase assays were performed in 96-well flat bottom plates (Costar 3596) and adapted from Schaefer et al. (2016). Colonies grown overnight on LB plates with appropriate antibiotics were used to inoculate LB. Log-phase culture, 80 μl each, was loaded in duplicates in a 96-well plate to which 120 μl of a custom mix (ONPG + Popculture reagent), prepared as described in (Schaefer et al., 2016), was added. Plates were incubated at 30°C in a Epoch2 plate reader (Biotek, United States) set to double orbital shaking and A_420_ measurements were taken in one- to 5-min intervals. Equivalent Miller Units (MU)were calculated using a Python program that parses OD_600_ and A_420_ values from the plate readers and plots A_420_ and ΔA_420_ values as a function of time, to identify maxima. Plotted β-galactosidase activity in MU represent means from three biological replicates and error bars depict SEM.

### Screen of Transposon-Insertion Mutants

The EZ-Tn5 transposome kit (Lucigen, WI) was used to generate random Tn insertions in *E. coli* DH10-β harboring pBJH235. The transformation mixtures were spread first on LB plates with appropriate antibiotics and incubated overnight at 37°C. The following day colonies were patched on MacConkey agar plates (MacConkey Agar Base [Difco, MD] supplemented with 1% (w/v) lactose and 3 mM 2-phenylethyl β-D-thiogalactoside (PETG, [Biosynth, IL], pH adjusted to 7.1). [PETG, a competitive inhibitor of β-galactosidase, was titrated to 3 mM, the concentration at which colonies with 75 MU appear white and those with 180 MU appear red (**Supplementary Figure S1**)]. Plates were incubated for 16 h at 37°C and colonies were monitored for development of red color. Candidate colonies were grown in LB overnight for genomic DNA isolation. Genomic DNA was extracted using the DNeasy Blood and Tissue Kit (Qiagen, CA, United States), digested with EcoRV-HF (NEB, MA, United States), and ligated overnight at 16°C with T4 DNA ligase (NEB). The ligation product was used to transform DH5α(λ*pir*) cells to recover transposon containing circularized genomic DNA, which were replication competent by virtue of the presence of R6Kγ*ori* within the transposon. Plasmid DNA was extracted from individual colonies using the QIAprep Spin Miniprep Kit (Qiagen) and sequenced using the primers supplied in the EZ-Tn5^TM^ transposome kit to identify the locations of Tn insertion.

### Deletion of *lrp* in *E. coli*

Δ*lrp-787::kan* was transduced from *E. coli* JW0872-2 into *E. coli* DH10-β and BR8706 (constitutive *araE*) using P1*vir* (Miller, 1992). The *kan* cassette was excised by expressing Flp recombinase from pCP20 and subsequently curing the plasmid by overnight growth at 42°C (Datsenko and Wanner, 2000).

### Deletion of *lrp* in *V. cholerae*

Deletion of *lrp* from CVC3058 (derivative of El Tor N16961 with P1*parS* cloned at +40 kb on Chr2 for visualizing *ori2* as GFP-P1ParB foci) was performed in the presence of a plasmid carrying *E. coli* *lrp* (pJWD-2), by natural transformation with linear DNA amplified from pPC352 that contained a *zeocin* cassette flanked by 1 kb upstream and downstream homology sequences. pPC352 was assembled using four DNA fragments: 1 kb upstream homology (amplified from genomic DNA using primers PNC123 and PNC124), 1 kb downstream homology (amplified from genomic DNA using primers PNC121 and PNC122), *zeocin* cassette (amplified from pEM7-Zeo using primers PNC127 and PNC128) and the backbone (amplified from pEM7-Zeo using primers PNC125 and PNC126). Linear DNA used for natural transformation was amplified from pPC352 using primers PNC131 and PNC132. Deletion of *lrp* was confirmed by PCR. The plasmid pJWD-2 was cured by growing overnight in the absence of antibiotic and screening colonies that had lost antibiotic resistance, to generate strain CVC3286. The deletion was verified by whole genome sequencing.

### Purification of Lrp and MBP-RctB

Lrp was purified from plasmid pJWD-2 (Ernsting et al., 1993). 5 ml of overnight culture of *E. coli* BL21 containing pJWD-2 was used to inoculate 1 liter of LB supplemented with ampicillin and grown at 37°C. Protein expression was induced at an OD_600 *nm*_ of 0.8 by adding IPTG to the final concentration of 0.5 mM, and growth was allowed to continue for 2.5 h. The pellet was resuspended in PC Buffer [50 mM phosphate buffer (pH 7.4), 100 mM NaCl, 0.1 mM EDTA, 10 mM β-mercaptoethanol and 10% glycerol, (de los Rios and Perona, 2007)] supplemented with 1× protease inhibitor cocktail (Sigma-Aldrich, St. Louis, MO) and lysed by French press. The lysate was clarified by centrifugation for 1 h at 18,000 ×*g* before loading onto a Hitrap SP HP column (GE Healthcare Life sciences, Chicago, IL, United States), pre-equilibrated with PC Buffer. Lrp was eluted using a gradient of PC Buffer + 1 M NaCl. The fractions containing Lrp were purified further by cation exchange on a Mono S column (GE Healthcare Life sciences) equilibrated with Cat2 buffer (50 mM Hepes (pH 8.0), 1 mM EDTA, 0.2% Tween20, 5% Glycerol and 100 mM NaCl). Lrp was eluted using a gradient of Cat2 buffer + 1 M NaCl. MBP-RctB was purified as described previously (Jha et al., 2017).

### Electrophoretic Mobility Shift Assay (EMSA)

Interaction of purified Lrp with *crtS* was captured *in vitro* using EMSA. The 153 bp *crtS* was flanked by ∼100 bp of lambda DNA and amplified from pBJH170 using FAM-labeled primers RR202 and RR214. Non-specific DNA was amplified from pTVC243 using the same primers as above and contained only the 100-bp flanks. Truncated *crtS* constructs Δ3′*crtS*, Δ5′*crtS*, and Δ5′Δ3′*crtS* were amplified from pPC189, pPC225 and pPC009, respectively, using primers PNC77 and PNC78. Increasing amounts of Lrp protein were added to 20 μl reactions that contained 5 nM each of fluorescent probe and vector DNA, 20 mM Hepes (pH 7.4), 1 mM EDTA, 0.2% Tween20, 5% glycerol, 200 ng poly dI-dC, 1 mM dithiothreitol, 70 mM potassium glutamate and 4 mM magnesium acetate. Leucine was added at 10 mM, when desired. The reaction was incubated at room temperature for 10 min before loading on a 5% native polyacrylamide gel and electrophoresed at 12 V/cm in 0.5 × TBE. The gel was scanned using Typhoon FLA 9500 (GE Healthcare Life Sciences, MA, United States). The image was analyzed, and band intensities quantified using Fiji software (Schindelin et al., 2012). The percent DNA bound was plotted against concentration of protein and K_*D*_ values were obtained by performing non-linear regression analysis assuming one site specific binding using GraphPad Prism version 7.0 a (La Jolla, CA, United States). Following EMSA of *crtS* with Lrp and RctB, the super-shifted band was excised from the native polyacrylamide gel and presence of both proteins confirmed by mass spectrometry performed at the Collaborative Protein Technology Resource (CCR, NIH) as previously described (Jha et al., 2017).

### Measurement of Plasmid Copy Number

Copy number experiments were performed using either WT *E. coli* (BR8706, constitutive *araE*) or Δ*lrp E. coli* (derivatives of BR8706: CVC3260, Δ*lrp-787::*FRT-*kan*-FRT and CVC3274 Δ*lrp-787*). BR8706 and CVC3260 were transformed with pTVC11 (p*rctB*) and either pTVC243 (vector), pBJH170 (p*crtS*), or pBJH239 (p*crtS-10 m*). CVC3274 was transformed with pTVC11, pPC401 (p*lrp)*, and either pTVC243, pBJH170, or pBJH239. To maintain high levels of RctB, competent cells were grown in 0.2% arabinose before and after transformation with pTVC22 (p*ori2*). Cultures were inoculated at an OD_600 *nm*_ of 0.005 and grown at 37°C with shaking to an OD_600 *nm*_ of 0.2. Eight OD units were pelleted and used for plasmid isolation. Relative plasmid copy number was measured essentially as described (Das and Chattoraj, 2004) but normalized to pTVC11.

### Whole Genome Sequencing

Genomic DNA was extracted from 1 ml of cells grown overnight at 37°C in LB using DNeasy Tissue Kit (Qiagen, Hilden, Germany). DNA was sequenced on the Illumina MiSeq platform at the NCI CCR genomics sequencing core. 1–6 million reads were obtained for each sample, which were trimmed and mapped to a CVC3058 reference genome using the CLC Genomics Workbench (Qiagen). The reference genome for CVC3058 was constructed by *de novo* assembly.

## Results

### 5′ Terminal Sequences of *crtS* Are Important for Licensing Chr2 Replication in *V. cholerae*

**FIGURE 1:**
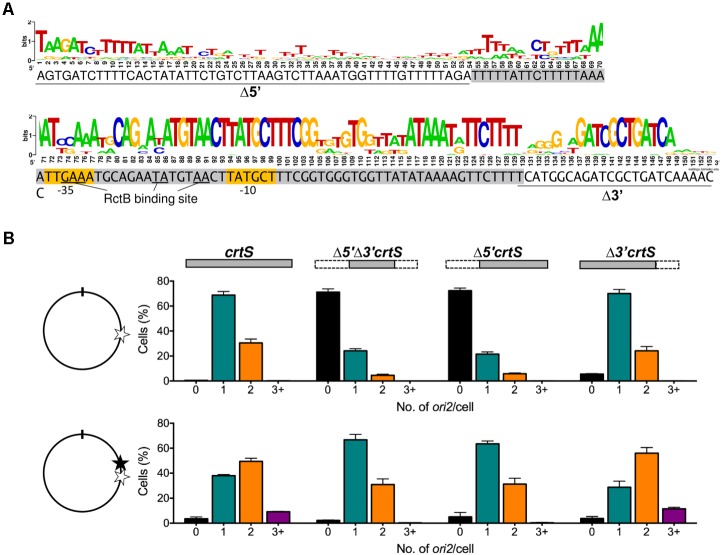
The 5′ but not the 3′ end of *crtS* is critical for its function. **(A)** The 153 bp *crtS* sequence from *V. cholerae* overlaid with a WebLogo (Crooks et al., 2004), generated from different *Vibrio* species showing varied conservation along the length of the sequence, as in Kemter et al. (2018). Gray highlighted sequence denotes the minimal 70-bp that retain copy-number enhancement function of *crtS* in *E. coli.* The flanking 5′ and 3′ sequences studied here are underlined. Yellow highlighted sequences denote the predicted –35 and –10 elements of the sigma-70 promoter, *P_crtS_*. **(B)** The effect of *crtS* and its truncated derivatives on Chr2 replication is shown by histograms of *ori2* foci numbers per cell in *V. cholerae* strains where *crtS* was replaced at its native locus with the truncated derivatives (top row), and where the same mutant strains had, at 10 kb upstream, a second full length *crtS* copy (bottom row). On the left of the histogram is shown the approximate location of *crtS* copies in Chr1, where the native locus is indicated by an empty star and the locus with the added *crtS* copy by a filled star. The position of the *ori1* is denoted by a tick-mark. The strains used were: intact *crtS* (CVC3058, top; CVC3061, bottom), Δ*5*′Δ*3*′*crtS* (CVC3228, top; CVC3247, bottom), Δ*5*′*crtS* (CVC3227, top; CVC3246, bottom), Δ*3*′*crtS* (CVC3226, top; CVC3245, bottom). Note that deletion of the upstream 53 bases (Δ5′) severely compromises replication-triggering function of *crtS* as evidenced by the appearance of cells with zero *ori2* foci. Data represent mean ± SEM (standard error of mean) of at least 1000 cells imaged from three biological replicates.

In *E. coli*, the presence of a plasmid containing 153 bp of *V. cholerae* Chr1 [coordinates 817947 to 818099 bp of Heidelberg et al. (Heidelberg et al., 2000)] (**Figure 1A**) increases the copy number of *ori2*-containing plasmids (p*ori2*) about threefold in the presence of RctB (Baek and Chattoraj, 2014). The 153 bp sequence was called *crtS* (Val et al., 2016). The central 54 - 123 bp, called Δ*5*′Δ*3*′*crtS* here, also increases the p*ori2* copy number about twofold in *E. coli* (Baek and Chattoraj, 2014). To test if Δ*5*′Δ*3*′*crtS* was sufficient to support replication of Chr2 in *V. cholerae* as well, the *crtS* sequence in Chr1 was replaced with Δ*5*′Δ*3*′*crtS* using natural transformation. Chr2 replication was followed by visualizing GFP-P1ParB bound to the *P1parS* site inserted 40 kb away from *ori2*, as previously described (Ramachandran et al., 2018). We found that Δ*5*′Δ*3*′*crtS* replacement resulted in a loss of *ori2* foci in 70% of cells (**Figure 1B**, top panel). Truncation of the 5′ and 3′ sequences separately revealed that this functional deficiency is due to the 5′ truncation, as truncation of the 3′ did not significantly alter the foci distribution. This suggests that the 1–123 bp of *crtS* locus spanning chromosomal coordinates 817947 to 818069 is sufficient for licensing Chr2 replication, despite the low conservation of the 5′ bases (**Figure 1A**). Furthermore, the results were indistinguishable in the Δ5′ constructs, whether or not the 3′ region was present (**Figure 1B**, top row).

Deletion of *crtS* leads to suppressor mutations in *rctB* or fusion of Chr1 and Chr2 (Val et al., 2016). To avoid the selection of suppressors while replacing the native *crtS* locus with truncated species, we repeated the replacements in strains that also possessed a second functional copy of *crtS* 10 kb upstream of the native locus (Ramachandran et al., 2018). The presence of two full length copies of *crtS* causes over-replication of Chr2 (Val et al., 2016) (**Figure 1B**, bottom row). This over replication was not seen when the native *crtS* locus was replaced with Δ*5*′*crtS*. Replacement with Δ*3*′*crtS* did not alter *crtS* function, as the distribution of *ori2* foci was similar to that of cells with two intact *crtS* copies. In sum, although the exact bounds of *crtS* remain to be defined, it appears that the 5′ sequence of *crtS* is essential for licensing replication from *ori2.*

### The Promoter Within *crtS* Is Repressed

**FIGURE 2:**
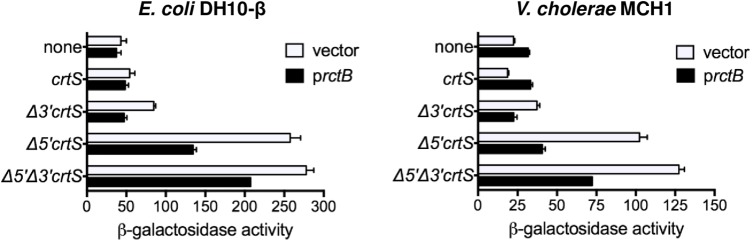
The promoter within *crtS* (*P_crtS_*) is repressed by an unknown factor common to *E. coli* and *V. cholerae.* β-galactosidase activity in *E. coli* DH10-β (left) and monochromosome *V. cholerae* MCH1 (right), containing promoterless *lacZ* in a pBR-based plasmid (none, pMLB1109), or *lacZ* transcriptionally fused to either *crtS* (pBJH235), Δ*3*′*crtS* (pPC066), Δ*5*′*crtS* (pPC067) and Δ*5*′Δ*3*′*crtS* (pPC068). Additionally, the strains had a second plasmid, p*rctB* (pRR24, black bars) supplying RctB or the corresponding empty vector (pPC020, white bars). The x-axis in the two graphs are scaled differently. Both in *E. coli* and MCH1, the promoter activity dramatically increases upon deletion of the 5′ *crtS* sequences. Since in both the strains the promoter repression is seen in the absence of RctB, the only factor known to bind *crtS*, an unknown factor common to two bacteria must be involved in repression of *P_crtS_*. Supplying RctB recovers the repression partially, which indicates that the promoter is normally repressed by RctB as well as the unknown factor. Error bars denote standard deviation of mean from three biological replicates.

In spite of the importance of the 5′ terminal sequences of *crtS*, they are not well conserved among the various *Vibrio* species (Kemter et al., 2018). Apart from AT-richness, the region does not have any known sequence features. *crtS*, however, possesses −35 and −10 promoter elements in the more conserved central region (**Figure 1A**). The promoter within *crtS*, called *P_crtS_* here, was previously shown to be expressed only from Δ*5*′Δ*3*′*crtS* but not from full length *crtS* (Baek and Chattoraj, 2014). From these results, it appears that the promoter repression and replication enhancement functions of *crtS* are correlated, and that the promoter repression may be necessary for *crtS* function. To quantify the promoter repression, we fused a promoterless *lacZ* gene to the *crtS* constructs used in **Figure 1B**. In *E. coli*, *P_crtS_* activity was as low as in the promoterless vector, but the activity increased fourfold in Δ*5*′*crtS* (**Figure 2**, left panel). These results indicate that an *E. coli* factor interacts with the 5′ terminal sequences of *crtS* and represses *P_crtS_.* A test of whether RctB, the only protein previously found to bind *crtS* (Baek and Chattoraj, 2014), could also repress the promoter showed that it did, but only partially (black vs. white bars, **Figure 2**). The expression of *P_crtS_* is thus controlled by at least two repressors. The deletion of the 3′ 30 bp had only a marginal effect on promoter activity.

In *V. cholerae*, truncation of the 5′ sequences results in only a slight increase in promoter activity (**Supplementary Figure S2**). To test whether the lack of increase could be due to the binding of *crtS* by RctB, the experiments were repeated in a strain of *V. cholerae*, MCH1, that lacks RctB and where Chr2 is maintained by fusion to Chr1 (Val et al., 2012). In MCH1, *P_crtS_* was expressed threefold higher in Δ*5*′*crtS* and Δ*5*′Δ*3*′*crtS* than *crtS*, mirroring the *E. coli* results (**Figure 2**, right panel). Addition of RctB caused partial repression of promoter activity in Δ*5*′*crtS* and Δ*5*′Δ*3*′*crtS*, as in *E. coli*. Together, these results strongly suggest that a factor other than RctB, common to both *E. coli* and *V. cholerae*, binds *crtS* and is responsible for the additional repression of the promoter within *crtS*.

### *P_crtS_* Is Repressed by the Global Regulator Lrp in *E. coli* and *V. cholerae*

**FIGURE 3:**
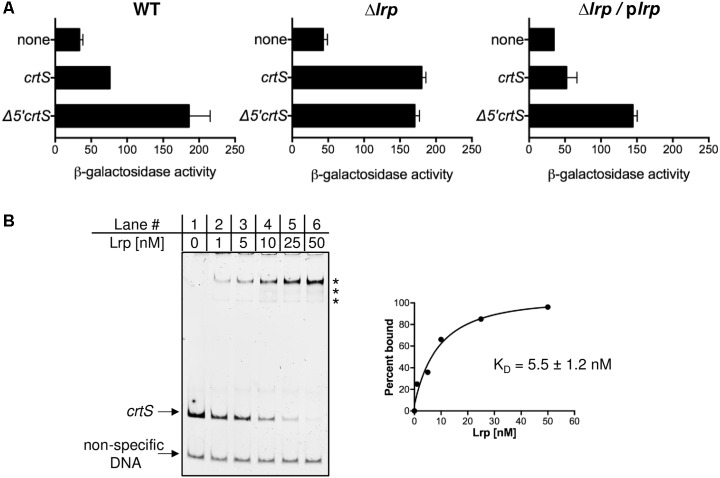
*P_crtS_* is repressed by the global regulator Lrp. **(A)** Promoter activity was measured after fusion to *lacZ* in WT (DH10-β), in Δ*lrp* (CVC3259) which is otherwise isogenic, and the same Δ*lrp* strain complemented with p*lrp* (CVC3259/pPC401). The *crtS* fragments were the same as in **Figure 2**. The deletion of *lrp* results in promoter de-repression, which is complemented in the presence of p*lrp* when *crtS* was full length and not when it was Δ5′. Error bars denote standard error of mean from two biological replicates. **(B)** Electrophoretic mobility shift assay (EMSA) of fluorescently labeled non-specific DNA (bottom arrow) and *crtS* DNA (top arrow) in the presence of increasing concentrations Lrp protein. Asterisks indicate positions of Lrp bound species. The fraction of the probe bound (quantified from the loss of intensity of the unbound probe) was plotted as a function of Lrp concentration to generate the binding isotherm that yielded an apparent dissociation constant (K_*D*_) of 5.5 ± 1.2 nM.

The putative *E. coli* factor responsible for repressing *P_crtS_* was identified by performing a transposon (Tn) insertional mutagenesis screen in strains that contained a plasmid with transcriptional-fusion of *crtS* to *lacZ*. Colonies with higher *lacZ* activity were identified by plating on MacConkey agar supplemented with 3 mM PETG, an inhibitor of β-galactosidase, that allowed clearer distinction between red and white colonies (Golding et al., 1991) (**Supplementary Figure S1**). In most of these colonies, the Tn was found to have inserted into the plasmid expressing *lacZ*. In one colony, the Tn was found to have inserted within the 5′ untranslated region of the *lrp* gene. To determine whether Lrp is responsible for the observed repression of *P_crtS_*, the promoter activity was measured in a *E. coli* Δ*lrp* strain [from Keio collection, (Baba et al., 2006)] and, although *crtS* was full length, the activity was as high as from Δ*5*′*crtS* (**Figure 3A**). Complementing the Δ*lrp* strain with Lrp using plasmid pJWD-2 (Ernsting et al., 1993) resulted in repression of *P_crtS_*, when present in intact *crtS* but not when present in Δ5′*crtS*. These results are fully consistent with Lrp being the factor that, directly or indirectly, keeps *P_crtS_* repressed.

An *in vitro* experiment was performed to test whether Lrp itself binds to *crtS*. *E. coli* Lrp protein was purified from plasmid pJWD-2 to about 95% purity. The *E. coli* protein is 92% identical to the *V. cholerae* Lrp protein and is completely conserved in the helix-turn-helix motif (Lintner et al., 2008). In EMSA using fluorescently labeled *crtS*, Lrp was seen to bind *crtS* with an approximate K_*D*_ of 5.5 ± 1.2 nM (**Figure 3B**). The addition of leucine altered the distribution of the Lrp-shifted species, indicating that *crtS-*binding is responsive to the presence of leucine (**Supplementary Figure S3**). Lrp was seen to bind equally well to Δ*3*′*crtS* and Δ*5*′*crtS* and slightly less well to Δ*5*′Δ*3*′*crtS*, indicating that it has multiple binding sites within *crtS*, but it appears that the site(s) within the 5′ terminal sequences are required for promoter repression (**Supplementary Figure S4**). A search for Lrp binding site within *crtS* using a SELEX derived consensus sequence (Cui et al., 1995) revealed a putative site with 12/15 matches covering 50 - 64 bp region. The first four bp of this putative Lrp binding site are lost upon truncation of the 5′ sequences, possibly explaining the loss of repression in Δ*5*′*crtS*. In addition to the 15 bp consensus, the three to five flanking bases also contribute to specific binding by Lrp (Cui et al., 1995), which are also missing in Δ*5*′*crtS.*

### Lrp Is Required for Chr2 Replication-Licensing by *crtS* in *E. coli* and *V. cholerae*

**FIGURE 4:**
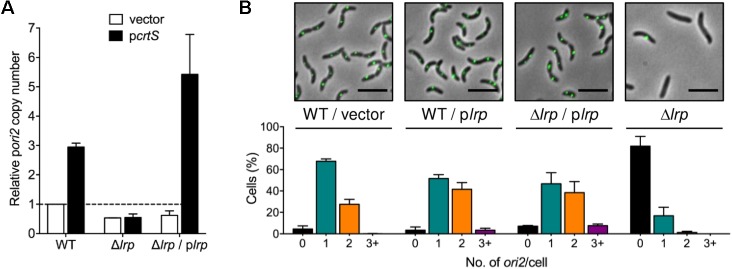
Lrp is necessary for licensing of Chr2 replication by *crtS*. **(A)** Copy number of p*ori2* (a plasmid carrying the origin of Chr2, pTVC22) in *E. coli* WT (BR8706), Δ*lrp* (CVC3260), and Δ*lrp/*p*lrp* (CVC3274/pJWD-2). The cells also contained a source of RctB (pTVC11) and additionally either a vector (white bars) or a *crtS*-containing plasmid (black bars). The copy numbers were normalized to the value in WT cells, set as 1. p*ori2* copy number increases threefold in the presence of p*crtS* in WT but not in Δ*lrp* cells. Complementing Δ*lrp* cells with p*lrp* increases p*ori2* copy number even more that that was seen in WT cells, suggesting that Lrp could be limiting in WT cells. Data represent mean ± SEM from three biological replicates. **(B)** Histograms of *ori2* foci number per cell in WT *V. cholerae* (CVC3058) with either the vector control (pTrc99A) or p*lrp*, and in *V. cholerae* Δ*lrp* cells (CVC3286) with complementing p*lrp* and after curing p*lrp.* Representative microscopy images from each strain are shown above the histograms. Note that the number of cells without any *ori2* foci increases in the absence of *lrp*, indicating that Lrp is crucial for Chr2 replication in *V. cholerae*. Data represent mean ± SEM from at least 1000 cells imaged from three biological replicates.

In order to test the effect of Lrp on the replication enhancement function of *crtS*, the copy number of *ori2-*containing plasmids was measured in Δ*lrp* strains. While in WT *E. coli*, the copy number of p*ori2* increased about threefold in the presence of p*crtS* compared to the empty vector, no such increase was observed in the Δ*lrp* strain (**Figure 4A**). This indicates that *crtS* fails to function as an enhancer of Chr2 replication in the absence of Lrp. Upon complementing with an Lrp-expressing plasmid, p*lrp*, the copy number of p*ori2* increased about fourfold in the presence of *P_crtS_*, whereas the vector copy number was unaffected, indicating that Lrp is essential for *crtS* function in *E. coli*. To test whether the Lrp was required solely to repress *P_crtS_*, a promoter-defective mutant of *crtS* (*crtS-10m*, (Baek and Chattoraj, 2014)) was used in which two bases within the −10 element of the promoter were mutated. The mutation was previously shown to retain the replication enhancement function of *crtS* in WT *E. coli* while possessing low promoter activity. However, p*crtS-10m* failed to increase p*ori2* copy number in the *E. coli* Δ*lrp* strain (**Supplementary Figure S5**). Introduction of the p*lrp* plasmid restored the function of p*crtS-10m.* This indicates that keeping the promoter repressed may not be the sole and perhaps not the primary function of Lrp on *crtS*.

Lrp is not essential for viability of *E. coli* or *V. cholerae* (Lintner et al., 2008; Srivastava et al., 2011; Fu et al., 2013). To test the requirement of Lrp for Chr2 replication in *V. cholerae*, the *lrp* gene was deleted in a strain where fluorescently tagged *ori2* foci could be visualized. The deletion was initially made in the presence of a plasmid supplying Lrp (pJWD-2). Upon deletion of chromosomal *lrp* and curing of p*lrp*, the percentage of cells without an *ori2* focus increased dramatically (from 7 to 80%) (**Figure 4B**). This indicates that although *lrp* gene is not essential, the protein contributes dramatically to Chr2 replication. The contribution seems greater in the defined medium used for microscopy, where the growth was slower than in LB (**Supplementary Figure S6**). In fact, the Δ*lrp* strain never appears to enter logarithmic growth in the microscopy medium. At least in *E. coli*, an Lrp-associated minimal medium growth defect results largely from effects in nitrogen assimilation (Paul et al., 2007; van Heeswijk et al., 2013). The requirement of Lrp in Chr2 replication/cell growth thus exhibits media-dependency.

Interestingly, in cells where the complementing p*lrp* plasmid was not cured, Chr2 copy number was higher than when the cells had the empty vector (**Figure 4B**). This outcome was obtained in the WT strain containing p*lrp* as well, where 45% of cells possessed two or more *ori2* foci as compared to 30% when cells contained the empty vector, suggesting that Lrp may normally be limiting for Chr2 replication. A test of whether Lrp functions solely via *crtS* to increase Chr2 replication was performed using a previously isolated Δ*crtS* strain (Baek and Chattoraj, 2014). This Δ*crtS* mutant possesses a mutation in *rctB*, which makes it a hyper-initiator (in WT *V. cholerae*) and apparently can compensate for the Chr2 replication defect that the absence of *crtS* confers. In this Δ*crtS* strain, p*lrp* failed to increase Chr2 replication (**Supplementary Figure S7**) and the distribution of *ori2* foci in Δ*crtS* with p*lrp*, resembled that of the vector control, indicating that Lrp functions via *crtS* in licensing Chr2 replication.

### Lrp Enhances RctB Binding to *crtS*

**FIGURE 5:**
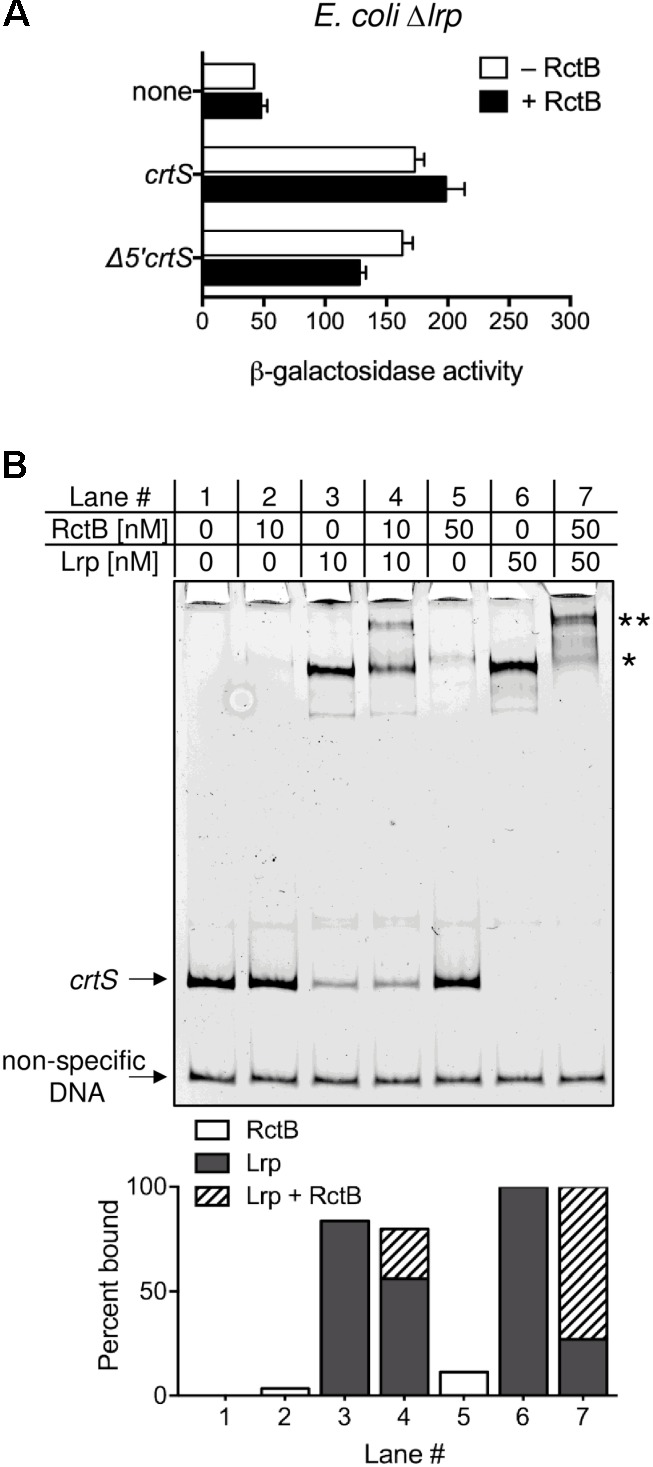
Lrp enhances RctB binding to *crtS*. **(A)** Promoter activity of *crtS* (from *P_crtS_*) was measured as in **Figure 2** in *E. coli* Δ*lrp* (CVC3259) carrying either the empty vector (none, pMLB1109), or the same vector carrying *crtS* (pBJH235) or its 5′ truncated derivative Δ*5*′*crtS* (pPC067). Cells also had either a source of RctB (pRR24) or the corresponding empty vector (pPC020). Note that RctB, which was partially effective in repressing *P_crtS_* in WT, fails to repress the promoter in Δ*lrp*. Data represent mean ± SEM from three biological replicates. **(B)** EMSA of 5′-FAM labeled *crtS* DNA (upper arrow) and non-specific DNA (lower arrow) with Lrp and RctB. Both Lrp (lanes three and six) and RctB (lanes two and five) were individually seen to bind *crtS* specifically. Note that the major Lrp bound band (^∗^) is super-shifted in the presence of RctB (^∗∗^). The intensity of the super-shifted band is much higher than the RctB bound band, indicating that RctB binds better to Lrp bound *crtS*. Shown below are percentages of probe bound to RctB alone (white columns), Lrp alone (gray columns) and, both Lrp and RctB (hatched columns).

How could Lrp stimulate *crtS* function? One possibility is that Lrp modulates the interaction of *crtS* with the rate-limiting factor for Chr2 replication, which is known to be RctB (Pal et al., 2005; Duigou et al., 2006). This hypothesis was tested *in vivo* and *in vitro*. In WT *E. coli*, RctB was able to repress the derepressed *P_crtS_* activity from Δ*5*′*crtS* by about 50% (**Figure 2**) but was unable to do so in the isogenic Δ*lrp* strain (**Figure 5A**). These results suggest that RctB binding to *crtS* is Lrp dependent. To test this hypothesis, we performed an EMSA of *crtS* DNA with both RctB and Lrp. RctB was previously shown to bind *crtS* only when the site is supercoiled (Baek and Chattoraj, 2014). However, by changing the buffer condition, it was possible to detect RctB binding to linear *crtS* fragments (**Figure 5B**). Lrp alone also bound to the same fragment but with more affinity. When both Lrp and RctB were present, a super-shifted band was seen, indicating that both proteins are bound to *crtS* simultaneously. Presence of RctB and Lrp in the super-shifted band was confirmed by mass spectrometry.

Leucine does not affect the binding of RctB to Lrp-bound *crtS* significantly (**Supplementary Figure S8**). While the total DNA bound by Lrp alone and by Lrp+RctB remained nearly the same, the intensity of the super-shifted band increased at the expense of the species bound to Lrp alone. Apparently, RctB binds with higher affinity to Lrp-bound *crtS* than to naked *crtS.* This is quantified by measuring RctB binding to either *crtS* or Lrp-bound *crtS* (**Supplementary Figure S9A**). The affinity of RctB to Lrp-bound *crtS* is nearly 10-fold higher than that to naked *crtS* (**Supplementary Figure S9B**). Lrp could also enhance the binding of RctB to Δ*5*′Δ*3*′*crtS* fragment (**Supplementary Figure S10**). This is not surprising, considering that Δ*5*′Δ*3*′*crtS* is functional in *E. coli* in multicopy, suggesting that RctB and Lrp can favorably interact on the Δ*5*′Δ*3*′*crtS* fragment, where at least one Lrp binding site also exists (**Supplementary Figure S4**). Lrp thus could enhance Chr2 replication by enhancing RctB binding to *crtS*.

## Discussion

### Requirement for Lrp in *crtS* Function

The Chr1 encoded *crtS-*mediated licensing of Chr2 replication is so far the only known mechanism by which the replication of one chromosome regulates the timing of the replication of the other. Here we report that the licensing function of *crtS* depends on the global transcription regulator, Lrp. Although many general DNA binding proteins, such as IHF, HU, Fis, and SeqA, are known to participate in DNA replication, this is the first evidence for Lrp participation, a protein sensitive to the environment and, in particular, to the intracellular concentration of leucine and other amino acids (Hart and Blumenthal, 2011). Growth phase control of DNA replication initiation is a little-studied aspect of cell cycle in bacteria, although starvation induced nucleotide alarmone (p)ppGpp has been known to inhibit new rounds of replication initiation for some time (Chiaramello and Zyskind, 1990). In the *E. coli* chromosome, the growth-phase regulated Fis protein signals to *oriC* to turn off DNA replication as the bacteria enter stationary phase (Cassler et al., 1995). So far, no Fis involvement in the origin of Chr2 replication has been detected in *V. cholerae*. The involvement of Lrp in Chr2 replication mirrors the involvement of Fis in sensitizing the chromosome to changes in cell physiology. The involvement of Lrp also makes *crtS* more comparable to DARS2 (DnaA reactivation site) of the *E. coli* chromosome (Kasho et al., 2014). Both *crtS* and DARS2 are involved in initiator remodeling, and both bind the cognate initiator and an additional factor, Lrp and Fis, respectively. The role of Lrp in *crtS* function thus could be analogous to Fis in DARS2 function.

We find that increasing Lrp concentration increases Chr2 replication (**Figure 4B**). Lrp concentration increases in stationary phase and upon other stresses to the cell (Landgraf et al., 1996). This suggests that Lrp could be utilized to promote Chr2 replication preferentially under stressful conditions. It is not possible to specify how the cells benefit from this preferential replication since functions of most of the genes in Chr2 are not known. It is known, however, that many more Chr2 genes are expressed during intestinal growth than during liquid culture, the basis of which is yet to be understood (Xu et al., 2003). One function of Lrp at *crtS* may be to maintain parity of chromosome numbers in stationary phase. In rich medium, Chr1 is maintained at two-fold higher copy number than Chr2 (Srivastava and Chattoraj, 2007; Stokke et al., 2011). When cells reach stationary phase both chromosomes have one copy each. To make this adjustment Chr2 must replicate an additional round after Chr1 replication has ceased. Increased Lrp concentrations during entry to stationary phase could help achieve this parity by stimulating Chr2 replication via *crtS*.

Lrp has been reported to control more genes in *E. coli* than any other global transcriptional regulators (Kroner et al., 2018; Shimada et al., 2018). A deletion of *lrp* in *E. coli*, however, is easily tolerated. In contrast, a deletion of *lrp* in *V. cholerae* is obtained only in the presence of a complementing plasmid and the deleted strain shows significant growth defect (Srivastava et al., 2011). Tn-seq analysis also showed fewer hits in *lrp* compared to many other targets considered “non-essential” in *V. cholerae* (Fu et al., 2013). The requirement of Lrp in *crtS* function, and hence in Chr2 replication, may explain why in *V. cholerae* Lrp is critical. Whole genome sequencing of Δ*lrp* strains cured of complementing plasmids in this study did not reveal any suppressor mutations in 2/2 cases. At least in our growth conditions (in LB), the Δ*lrp* strains appear to be viable, although slow-growing, whereas growth is more severely affected in the poorer synthetic medium used for microscopy (**Supplementary Figure S6**). *V. cholerae* possesses three hypothetical genes with significant identity (>35%) to Lrp (**Supplementary Table S4**), in addition to the widely distributed local regulator AsnC (Caspi et al., 2016; Unoarumhi et al., 2016). It is possible that in the absence of Lrp, some of its functions could be compensated for by these paralogs. If any paralogs exist in *E. coli*, they do not seem to substitute for Lrp. In *E. coli* the protein seems to be essential for *crtS* function (**Figure 4A**).

### The Importance of the Less-Conserved 5′ Region of *crtS*

An intriguing feature of *crtS* is that its 5′ region, although less conserved than the remainder of the site, is crucial for its replication enhancement function. On the other hand, that same function is unaffected by deletion of the downstream sequences, which are better conserved. The conservation of non-essential region suggests that *crtS* serves additional functions that are not yet recognized. Variant forms of Lrp or its orthologs in different species may account for the relatively poor conservation of the 5′ region. If so, this likely involves the differences at the amino termini of the different *Vibrio* Lrp orthologs (Hart et al., 2011; Unoarumhi et al., 2016). Although *crtS* sequences from different *Vibrio* species are able to increase the copy number of orthologous p*ori2*, the failure of certain *crtS* sequences to function with a few other p*ori2*, could be due to differences in their cognate Lrp proteins (Kemter et al., 2018).

The 5′ region of *crtS* provides Lrp binding sites required for promoter repression as well as the enhancement of replication initiation. The nature of the relationship of the two functions to each other remains to be clarified, but they appear to be anti–correlated: truncation that resulted in increased promoter expression reduces the efficiency with which *crtS* can license Chr2 replication. It is possible that occupancy by RNA polymerase interferes with RctB binding to *crtS.* The presence of Lrp could thus aid RctB binding to *crtS* by preventing RNA polymerase from binding to the promoter. Lrp usually forms an octameric ring composed of two tetramers, upon which DNA is wrapped, causing significant bending to the DNA (de los Rios and Perona, 2007). It is possible that the bases on *crtS* preferred by RctB are made more accessible by bound Lrp, or that constructive protein-protein contacts are made between Lrp and RctB.

The low *P_crtS_* activity under our laboratory conditions measured with a transcriptional fusion to *lacZ* was also evident from previous RNA-Seq analyses (**Figure 2**) (Baek and Chattoraj, 2014; Papenfort et al., 2015). RNA-Seq reads in *V. cholerae* at low and high cell densities did not reveal any measurable transcripts originating from *P_crtS_*. Unless some conditions are found that activate the promoter naturally, the presence of the promoter might well be incidental to the Lrp requirement in *crtS* function (Alice and Crosa, 2012; Lin et al., 2007). Uncoupling the replication enhancement and promoter repressor function of Lrp by mutating the −10 box of *P_crtS_* did not relieve the site from Lrp dependence (**Supplementary Figure S5**). This indicates that reduction of the promoter activity cannot be the only role of Lrp. To the extent analyzed, increasing RctB binding appears to be the main function of Lrp. In the immediate future, we seek to delineate the details of interactions among *crtS*, RctB and Lrp with the ultimate aim of understanding how they help RctB to license Chr2 replication and regulate that essential function.

## Author Contributions

PC, RR, and DC designed the study and wrote the manuscript. PC and RR performed the experiments. All authors read and approved the final version.

## Conflict of Interest Statement

The authors declare that the research was conducted in the absence of any commercial or financial relationships that could be construed as a potential conflict of interest.
