# Supplementary material for: A Requirement for Global Transcription Factor Lrp in Licensing Replication of Vibrio cholerae Chromosome 2
Source: Front Microbiol. 2018 Sep 10;9:2103. doi: 10.3389/fmicb.2018.02103 (PMC6139311; doi:10.3389/fmicb.2018.02103)
Supplement: Supplementary file 1 [file Data_Sheet_1.pdf]

Supplementary Material

A Requirement for Global Transcription Factor Lrp in Licensing Replication of *Vibrio cholerae* Chromosome 2

Peter N. Ciaccia[1], Revathy Ramachandran[1*], Dhruba K. Chattoraj[1]

*** Correspondence:** Revathy Ramachandran, revathy.ramachandran@nih.gov

Supplementary Figures

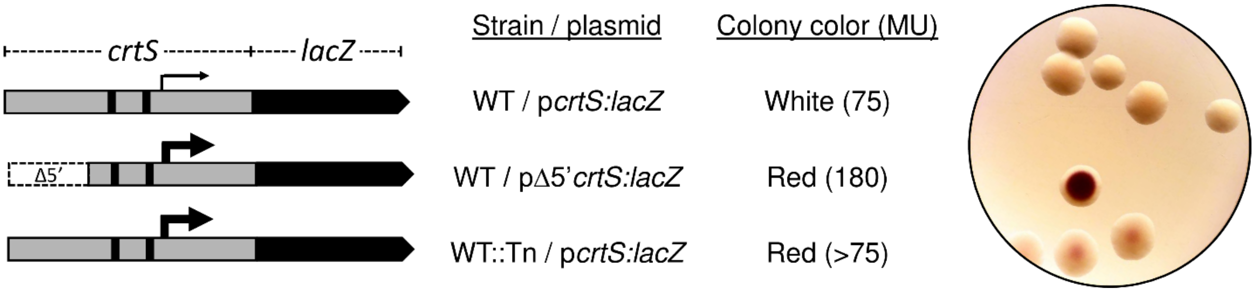

Figure S1. A scheme for identification of factors that repress P_{crtS}. (A) Transposon (Tn) insertional mutagenesis screen in *E. coli* DH10-β that contained a plasmid with transcriptional-fusion of *crtS* to a promoter-less *lacZ* gene (pBJH235). The -35 and -10 elements of the promoter in *crtS*, P_{crtS}, are shown by black bars. On MacConkey agar supplemented with 3 mM PETG, the promoter activity is low enough that the cells make white colonies. When the 5' sequences of *crtS* are deleted, the colonies become red due to increased activity of P_{crtS}. Our premise is that inactivation of chromosomal genes (by transposon insertion) that are involved in repression of P_{crtS} will increase the promoter activity similar to the level seen with Δ*5'crtS*, and colonies of such transposon carrying cells can be recognized by their red color. The color difference is displayed here by 1:10 mixture of cells containing *crtS* vs. Δ*5'crtS* fused to promoter-less *lacZ* gene. β-galactosidase activities are in Miller units (MU).

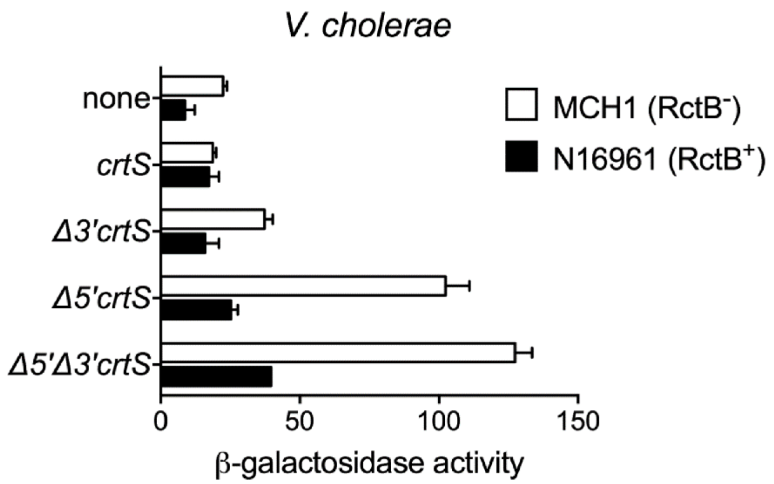

Figure S2. *P*crtS is repressed in *V. cholerae.* β-galactosidase activity in *V. cholerae* N16961 (black columns) either containing a promoterless *lacZ* in a pBR-based plasmid (none, pMLB1109), or the same *lacZ* transcriptionally fused to either *crtS* (pBJH235), Δ*3'crtS* (pPC066), Δ*5'crtS* (pPC067) and Δ*3'*Δ*5'crtS* (pPC068). For comparison, β-galactosidase activities in MCH1 from Figure 2 are reused here (white columns). Note that unlike in *E. coli* and MCH1 (Figure 2), the promoter activity increases marginally upon deletion of the 5' *crtS* sequences, presumably because of the chromosomal source of RctB, which can bind and repress *P*crtS. Data represent mean ± SEM from three biological replicates.

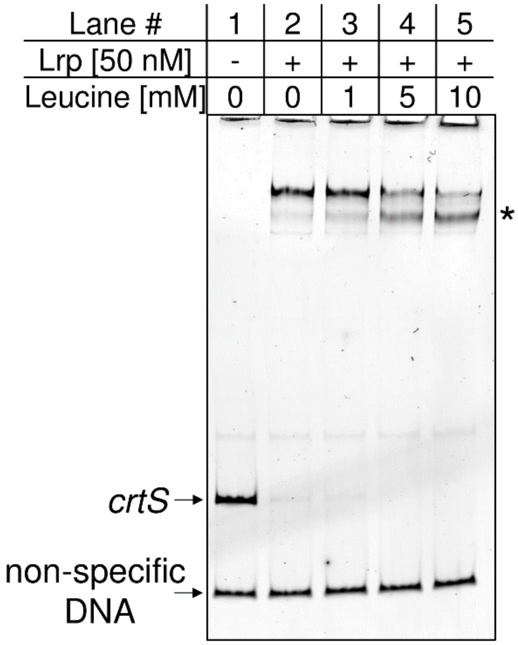

Figure S3. Leucine alters Lrp binding to *crtS*. EMSA of fluorescently labeled *crtS* (upper arrow) and non-specific DNA (lower arrow) with 50 nM Lrp protein in presence of increasing concentrations of leucine. With increasing leucine, the intensity of the middle-retarded band (*) increases at the expense of the upper retarded band, indicating that the Lrp binding is responsive to leucine.

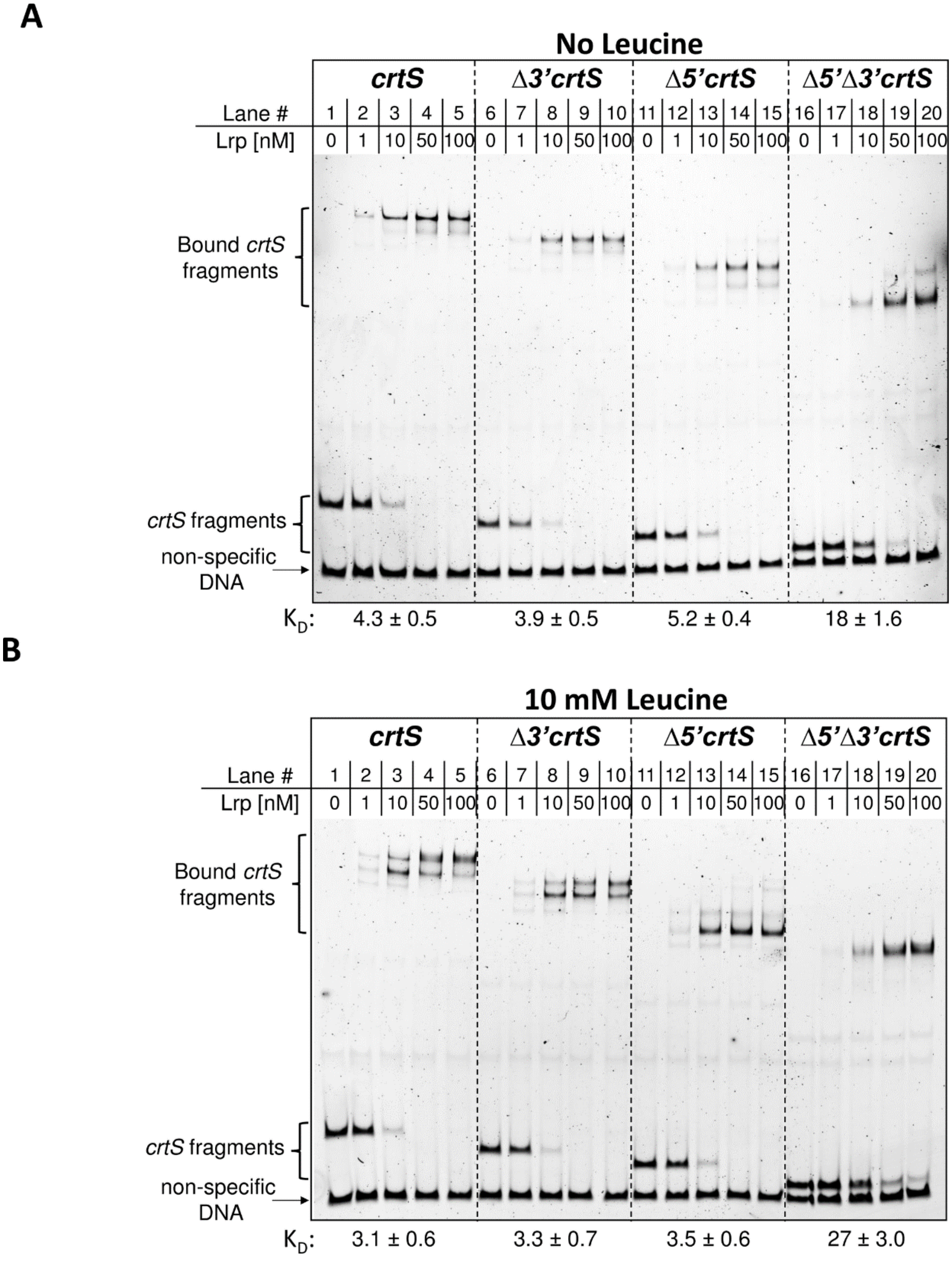

Figure S4. Lrp binds to multiple sites on *crtS*. (A) EMSA of fluorescently labeled *crtS*, Δ*3'crtS*, Δ*5'crtS* and Δ*5'*Δ*3'crtS* with increasing amounts of Lrp protein. Lrp binds to all three fragments but with distinct patterns, indicating the presence of multiple binding sites within *crtS*. Apparent dissociation constant (K_D) calculated from two trials is shown below each DNA fragment. **(B)** EMSA performed as described above but in the presence of 10 mM leucine. Presence of leucine did not change the fraction of the probe bound but altered the pattern of shifted species, as in Figure S3.

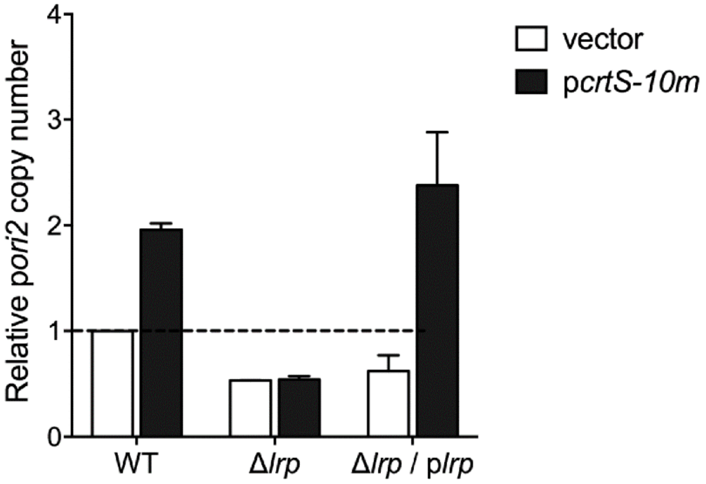

Figure S5. Promoter repression is not the only role of Lrp in Chr2 replication. Copy number of p*ori2* in *E. coli* WT, Δ*lrp*, and Δ*lrp/*p*lrp*. The cells also contained a source of RctB and additionally either a vector (white columns, pTVC243) or a plasmid containing *crtS-10m* (black columns, pBJH239), where two bases in the -10 are mutated (TATGCT to CCTGCT), that inactivates P_{crtS}. The other details are same as in Figure 4A. Note that p*ori2* copy number increases two-fold in the presence of p*crtS-10m* in WT but not in Δ*lrp* cells, indicating that Lrp is required to promote Chr2 replication even when P_{crtS} is not active. Complementing Δ*lrp* cells with p*lrp* increases p*ori2* copy number even more that that was seen in WT cells, similar to the effect seen in Figure 4A with p*lrp.* Data represent mean ± SEM from three biological replicates.

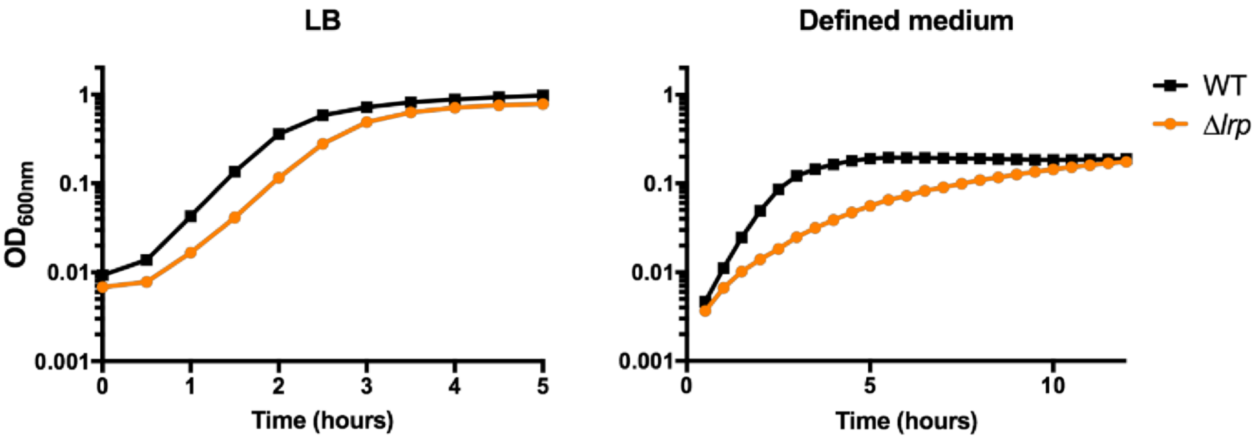

Figure S6. ***V. cholerae* Δ*lrp* has less growth defect in rich medium (LB) than in a less rich defined medium used here for fluorescence microscopy.** Growth curve of *V. cholerae* Δ*lrp* (orange circles, CVC3286) and WT (black squares, CVC3058) in LB or 1X M63 medium supplemented with 1 mM $CaCl_2$, 1 mM $MgSO_4$, 0.001% vitamin B1, 0.2% fructose and 0.1% casamino acids, showing a larger growth defect in the latter medium. Cells were grown at 37 °C in 96-well plates in a Synergy HT plate reader (Biotek). Data represents mean from two biological replicates.

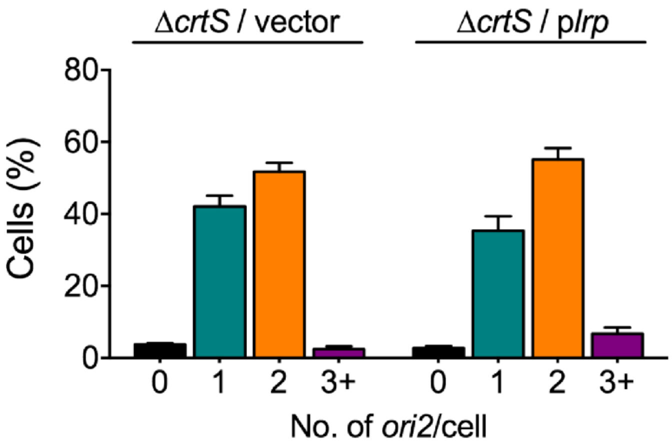

Figure S7. Lrp functions via *crtS* in increasing Chr2 replication in *V. cholerae.* Histograms of *ori2* foci per cell in *V. cholerae* Δ*crtS* (CVC1270) with either vector (pTrc99A, left) or p*lrp* (pJWD-2, right). The Δ*crtS* strain contains a mutation in *rctB* that permits Chr2 replication in the absence of *crtS*. Unlike in the WT (Figure 4), increasing Lrp using p*lrp* fails to increase number of *ori2* foci in the Δ*crtS* strain, indicating that Lrp promotes Chr2 replication only via *crtS.* Data represent mean ± SEM of at least 1000 cells imaged from three independent replicates.

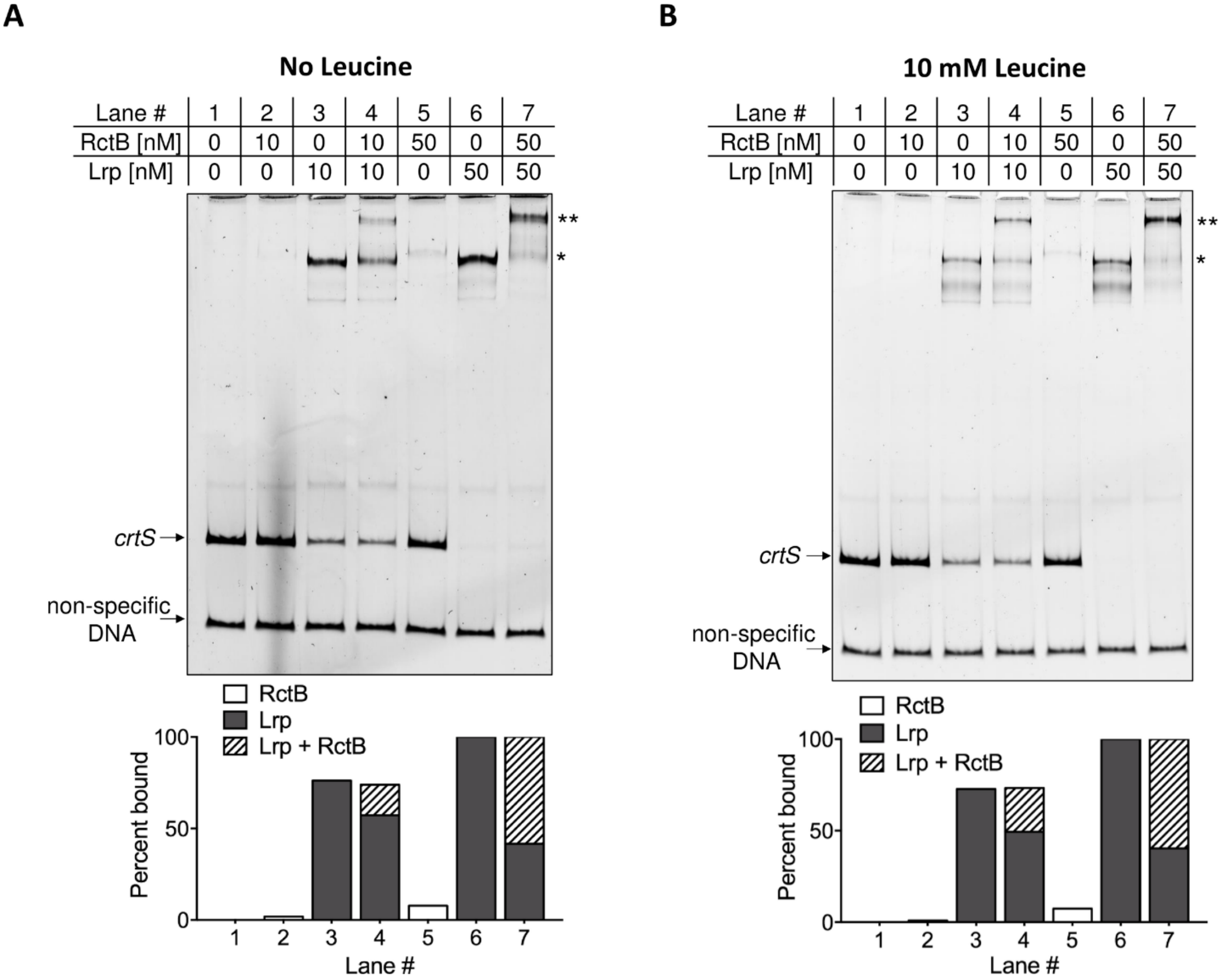

Figure S8. Lrp-aided RctB binding to *crtS* is not affected in the presence of Leucine. EMSA of fluorescently labeled *crtS* with Lrp, RctB or both proteins in the absence **(A)** or presence of 10 mM leucine **(B)**. The Lrp-bound *crtS* is denoted by *, and Lrp+RctB bound *crtS* is denoted by **. Leucine does not affect the binding of RctB to Lrp-bound *crtS* significantly. Shown below are percentages of probe bound to RctB alone (white columns), Lrp alone (grey columns) and, both Lrp and RctB (hatched columns).

A

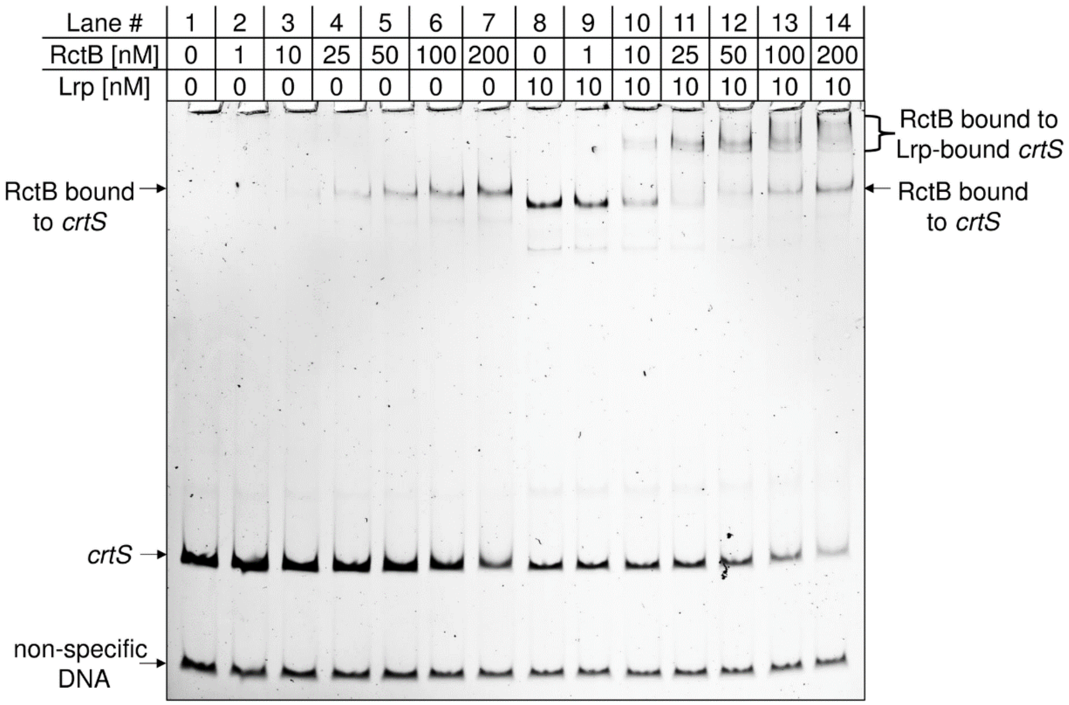

B

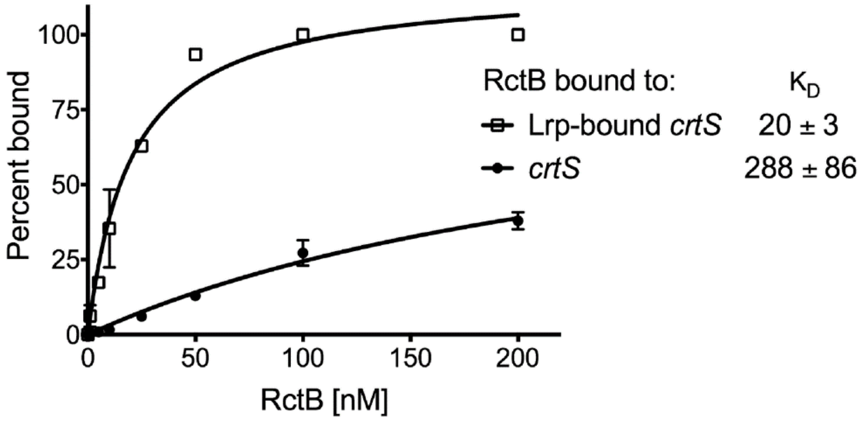

Figure S9: RctB has higher affinity for Lrp-bound *crtS*. (A) EMSA of fluorescently labeled *crtS* with increasing amounts of RctB in the absence (lanes 2-7) or presence of 10 nM Lrp (lanes 9-14), showing that RctB binds to Lrp-bound *crtS* in a dose-dependent manner. RctB binds to naked *crtS* once all of the Lrp-bound *crtS* is bound. **(B)** Quantification of percent bound probe, defined as bound/(bound + unbound), plotted against RctB concentration. The K_D of RctB binding to Lrp-bound *crtS* is lower than that to *crtS* alone. The data is collected from two gels.

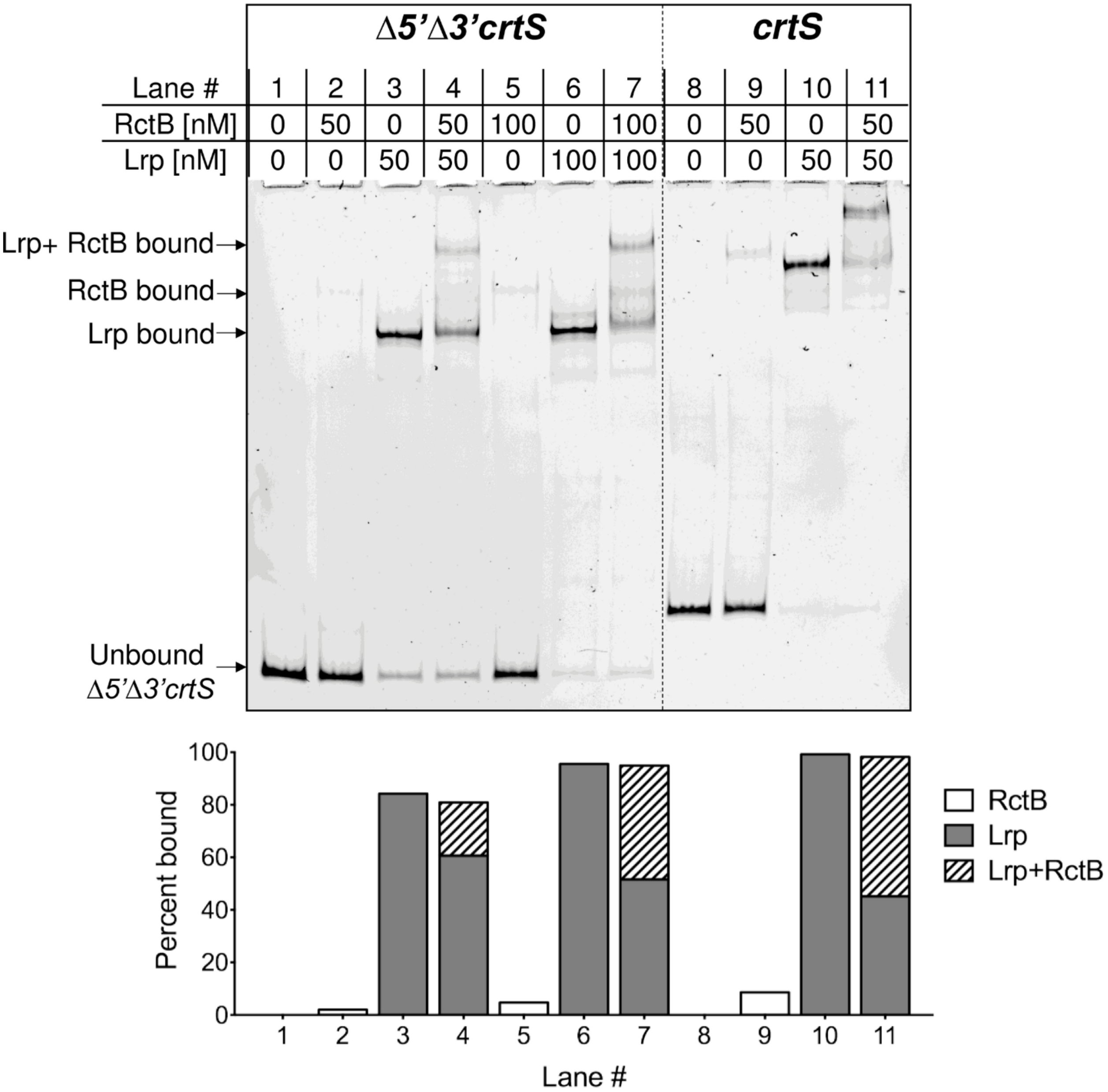

Figure S10: Lrp aids RctB binding to Δ*5'*Δ*3'crtS.* EMSA of fluorescently labeled Δ*5'*Δ*3'crtS* (lanes 1-7) and *crtS* (lanes 8-11) with Lrp, RctB or both proteins. Both Lrp (lanes 3, 6) and RctB (lanes 2, 5) were individually seen to bind DNA specifically, although RctB binds Δ*5'*Δ*3'crtS* weakly. The major Lrp-bound band is super-shifted in the presence of RctB for Δ*5'*Δ*3'crtS* as well as full length *crtS*. Shown below are percentages of probe bound to RctB alone (white columns), Lrp alone (grey columns) and, both Lrp and RctB (hatched columns).

Supplementary Tables

Table S1. Bacterial strains used in this study

Strain	Genotype	Description	Source
E. coli			
BL21	*E. coli* B F⁻ *ompT gal dcm lon hsdS$_B$*($r_B^-m_B^-$) [*malB*$^+$]$_{K-12}$(λ^S)		NEB, Ipswich, MA
BR8706	F$^-$, *mcrA*, Δ(*mcrBC-hsdRMS-mrr*), *recA1*, *endA1*, *lon*, *gyrA96*, *thi*, *supE44*, *relA1*, λ⁻, Δ(*lac-proAB*), Δ(*araFGH*), Δ*araEp* P$_{CP18}$-*araE*	Stbl2 with *araFGH* deleted and *araE* placed under the constitutive CP$_{18}$ promoter	(Fekete and Chattoraj, 2005)
CVC3258	DH10-β Δ*lrp-787::*FRT-*kan*-FRT*,* Km^R	Δ*lrp-787*::FRT-*kan*-FRT transduced from JW0872-2	This study
CVC3259	DH10-β Δ*lrp-787*	*kan* excised from CVC3258 with pCP20	This study
CVC3260	BR8706 Δ*lrp-787::*FRT-*kan*-FRT*,* Km^R	Δ*lrp-787*::FRT-*kan*-FRT transduced from JW0872-2	This study
CVC3274	BR8706 Δ*lrp-787*	*kan* excised from CVC3260 with pCP20	This study
DH10-β	F⁻ *endA1 deoR*$^+$ *recA1 galE15 galK16 nupG rpsL Δ(lac)X74* φ80*lacZΔM15 araD139 Δ(ara, leu)7697 mcrA Δ(mrr-hsdRMS-mcrBC)* Str^R λ⁻		Invitrogen
JW0872-2	F-, Δ*(araD-araB)567*, *ΔlacZ4787*(::*rrnB*-3), *λ*$^-$, *Δlrp-787::kan*, *rph-1*, *Δ(rhaD-rhaB)568*, *hsdR514,* Km^R	BW25113 *Δlrp*	(Baba et al., 2006)
V. cholerae			
CVC1121	N16961 *hapR*$^+$ Δ*dns*; Str^R, Gm^R	Frameshift mutation in *hapR* replaced with constitutively expressed *hapR*	M. Blokesch
CVC1270	CVC1121 Δ*crtS* P1*parS kan lacZ*::(*araC* P$_{BAD}$-*tus lacI*^{q} P$_{tac}$ -(*tdTomato*-pMT*parB gfp*-P1*parB t1t2*) *cat*) *rctB*R423P; Gm^R, Km^R, Cm^R	P1*parS* cloned at +40 kb on Chr2 for visualizing *ori2*	This study
CVC209	*V. cholerae* El Tor N16961; Str^R	Wild type	M. Waldor

CVC3058	CVC1121 P1*parS lacZ*::(*araC* P_{BAD}-*tus lacI*^{q} P_{tac}-(*tdTomato*-pMT*parB gfp*-P1*parB t1t2*), *cat*); Gm^R, Cm^R	P1*parS* cloned at +40 kb on Chr2 for visualizing *ori2*	(Ramachandran et al., 2018)
CVC3061	CVC3058 *crtS*-*nat*; Gm^R, Cm^R, Nat^R	A second copy of *crtS* inserted 10 kb upstream of WT copy	(Ramachandran et al., 2018)
CVC3226	CVC3058 Δ*crtS*:: Δ*3'crtS* (817947-818069) *zeo*; Cm^R, Gm^R, Km^R, Zeo^R	*crtS* replaced with Δ*3'crtS* mutant	This study
CVC3227	CVC3058Δ*crtS*:: Δ*5'crtS* (818000-818099) *zeo*; Cm^R, Gm^R, Km^R, Zeo^R	*crtS* replaced with Δ*5'crtS* mutant	This study
CVC3228	CVC3058Δ*crtS*:: Δ*5'*Δ*3'crtS* (818000-818069) *zeo*; Cm^R, Gm^R, Km^R, Zeo^R	*crtS* replaced with Δ*5'*Δ*3'crtS* mutant	This study
CVC3286	CVC3058 Δ*lrp zeo*	*lrp* replaced with zeocin resistance cassette	This study
MCH1	CVC209 Δ*lacZ* Δ(*parB2-rctB*)	Chr1 and Chr2 fused	(Val et al., 2012)

Table S2. Plasmids used in this study

Name	Description	Source
pACYC177	Vector; p15A*ori*; Ap^R, Km^R	NEB, Ipswich, MA
pBJH170	pTVC243 *crtS* (817947-818099); Cm^R	(Baek and Chattoraj, 2014)
pBJH188	pACYC177 Δ*bla crtS* (817947-818099); p15A*ori*; Km^R	(Baek and Chattoraj, 2014)
pBJH235	pMLB1109 *crtS* (817947-818099); Ap^R	(Baek and Chattoraj, 2014)
pBJH245	1 kb homology upstream and downstream of *crtS* with *zeo* in place of *crtS*; pBR*ori;* Zeo^R, Ap^R	(Baek and Chattoraj, 2014)
pCP20	Source of Flp recombinase; Ap^R, Cm^R	(Datsenko and Wanner, 2000)
pEM7-Zeo	Cloning vector and source of *zeo* cassette; pBR*ori*; Ap^R, Zeo^R	Invitrogen
pJWD-2	Source of Lrp; Ap^R	(Ernsting et al., 1993)
pMLB1109	Source of promoterless *lacZ* gene; Ap^R	M. Berman
pMT101::*recA*+	*recA* cloned in pSC101*rep*(ts); Cm^R, ts	G. Weinstock
pPC020	pACYC177 Δ*bla*; p15A*ori*; Km^R	This work
pPC066	pMLB1109 Δ*3'crtS* (817947-818069); Ap^R	This work
pPC067	pMLB1109 Δ*5'crtS* (818000-818099); Ap^R	This work
pPC068	pMLB1109 Δ*5'*Δ*3'crtS* (818000-818069); Ap^R	This work
pPC143	pBJH245 with Δ*3'crtS* (817947-818069) inserted between flanks; Ap^R, Zeo^R	This work
pPC144	pBJH245 with Δ*5'crtS* (818000-818099) inserted between flanks; Ap^R, Zeo^R	This work
pPC145	pBJH245 with Δ*5'*Δ*3'crtS* (818000-818069) inserted between flanks; Ap^R, Zeo^R	This work
pPC352	Upstream flank of *lrp* (2053847-2054879) and downstream flank of *lrp* (2056384-2055375) cloned into pEM7-Zeo on either side of *zeo*; Ap^R, Zeo^R	This work
pPC401	P_{trc} and *lrp* from pJWD-2 cloned into pACYC177; Km^R	This work
pRR24	RctB source in which *rctB* is transcriptionally fused to *kan* in pACYC177; p15A*ori*; Km^R	(Ramachandran et al., 2018)
pTrc99a	Vector; pBR*ori*; Ap^R	H. Bernstein
pTVC11	P_{BAD}-*rctB* in pGB2; pSC101*ori*; Sp^R	(Pal et al., 2005)
pTVC22	*ori2* (253-1133) in R6K*oriγ*, Ap^R	(Venkova-Canova et al., 2006)
pTVC243	Vector; pBR322*ori*; Cm^R	(Venkova-Canova and Chattoraj, 2011)

Table S3. Primers used in this study

Name	Sequence	Used for amplifying
PNC046	TATAGTGAAAAGATCACTCTAG AGGATCCCCGGGAA	pBJH245 backbone to construct pPC143
PNC047	TTCCCGGGGATCCTCTAGAGTG ATCTTTTCACTATA	Δ*3'crtS* from pBJH188 to construct pPC143
PNC050	CATTTCAATTTTAAAAAGAATA AAAACTAGAGGATCCCCGGGA	pBJH245 backbone to construct pPC144 and pPC145
PNC051	TCCCGGGGATCCTCTAGTTTTT ATTCTTTTTAAAATTGAAATG	Δ*5'crtS* and Δ*5'*Δ*3'crtS* from pBJH188 to construct pPC144 and pPC145
PNC053	AGATCGCTGATCAAAACCTTTT GAGCAGAAGATCCGG	pBJH245 backbone to construct pPC144
PNC054	GGTGGGTGGTTATATAAAAGTT TTTGAGCAGAAGATCCGG	pBJH245 backbone to construct pPC143 and pPC145
PNC055	CCGGATCTTCTGCTCAAAAGGT TTTGATCAGCGATCT	Δ*5'crtS* from pBJH188 to construct pPC144
PNC056	CCGGATCTTCTGCTCAAAAACT TTTATATAACCACCCACC	Δ*3'crtS* and Δ5'Δ3'*crtS* from pBJH188 to construct pPC143 and pPC145
PNC077	/56FAM/TTGAAGACGAAAGGGC CTC	DNA probes for EMSA from pACY177-based plasmids
PNC078	/56FAM/CCGCCTCCATCCAGTC	
PNC121	CGATTAAGTTGGGTAGCTCTGG TTTCAACTCGACTTTAGGCTCC ACTTG	1kb downstream flanking region of *lrp* from genomic DNA of CVC1121 to construct pPC352
PNC122	GCCGAGGAGCAGGACCTTAAA GCGAATGTTGGAGATCTTTCCA CATTCTGAC	
PNC123	AGGCCAGCAAAAGGTTTTTATT CCACCTTATTACTTCCTTGCAA AAAAATATACTACAACTTC	1kb upstream flanking region of *lrp* from genomic DNA of CVC1121 to construct pPC352
PNC124	CAGCAACGCGGCTCTGCGTGAG TGGTAGGGTGAGAGGTTTC	
PNC125	AGTTGAAACCAGAGCTACCCAA CTTAATCGCCTTGCAGCACATC	*bla* and pBR322*ori* in pEM7-*zeo* to construct pPC352
PNC126	TACCACTCACGCAGAGCCGCGT TGCTGGCGTTTTTCC	
PNC127	AGGTGGAATAAAAACCTTTTGC TGGCCTTTTGCTCACATGCTG	EM7 promoter and *zeo* gene in pEM7-*zeo* to construct pPC352
PNC128	CATTCGCTTTAAGGTCCTGCTC CTCGGCCACGAAGTG	
PNC131	GCTCTGGTTTCAACTCGACTTT AGGCTCCACTTG	Upstream flank, P_{EM7}-*zeo*, downstream flank from pPC352 to construct CVC3286
PNC132	TCTGCGTGAGTGGTAGGGTGAG AGGTTTC	

PNC139	GAACGTCGTGCGCTCACATGTTCTTTCCTGCGTTATCCCCTGATTCTG	*lacI*[q], P_{trc}*lrp* from pJWD-2 to construct pPC401
PNC140	TCTCAGCGATCTGTCTTAGCGCGTCTTAATAACCAGACGATTACTCTGC	
PNC141	AGACGCGCTAAGACAGATCGCTGAGATAGGTGCCTCACTG	pACYC177 backbone to construct pPC401
PNC142	ATGTGAGCGCACGACGTTCCTGTCCACGGTACGC	
RR160	ATGAGCATGGTGAAAAACCGC	Upstream flank, P_{EM7}-*zeo*, and downstream flank from pPC143, pPC144, and pPC145 to construct CVC3226, CVC3227, and CVC3228, respectively
RR162	GGAATTGGTCAGCCGTGATCAT	
RR202	/56FAM/ATCCGATTACGGCACCAAATCGA	DNA probes for EMSA from pTVC243-based plasmids
RR214	/56FAM/AACGTGGATAAACTTCCTGTAAT	

Table S4: Predicted paralogs of Lrp in *V. cholerae*

Lrp paralog	Accession number	Gene ID	% identity	Alignment length	e-value
Lrp	NP_231538.1	VC1904	100	164	2.94E-120
#1	NP_233450.1	VCA1068	45.517	145	2.88E-41
#2	NP_231349.1	VC1713	40.816	147	6.67E-33
#3	NP_230707.2	VC1062	35.135	148	1.32E-30
#4 (AsnC)	NP_229730.1	VC0071	24.828	145	1.94E-11

Supplementary References

Baba, T., Ara, T., Hasegawa, M., Takai, Y., Okumura, Y., Baba, M., Datsenko, K.A., Tomita, M., Wanner, B.L., and Mori, H. (2006). Construction of *Escherichia coli* K-12 in-frame, single-gene knockout mutants: the Keio collection. Mol Syst Biol *2*, 2006 0008.

Baek, J.H., and Chattoraj, D.K. (2014). Chromosome I Controls Chromosome II Replication in *Vibrio cholerae*. PLoS Genet *10*, e1004184.

Datsenko, K.A., and Wanner, B.L. (2000). One-step inactivation of chromosomal genes in *Escherichia coli* K-12 using PCR products. Proc Natl Acad Sci U S A *97*, 6640-6645.

Ernsting, B.R., Denninger, J.W., Blumenthal, R.M., and Matthews, R.G. (1993). Regulation of the *gltBDF* operon of *Escherichia coli*: how is a leucine-insensitive operon regulated by the leucine-responsive regulatory protein? J Bacteriol *175*, 7160-7169.

Fekete, R.A., and Chattoraj, D.K. (2005). A cis-acting sequence involved in chromosome segregation in *Escherichia coli.* Mol Microbiol *55*, 175-183.

Pal, D., Venkova-Canova, T., Srivastava, P., and Chattoraj, D.K. (2005). Multipartite regulation of *rctB*, the replication initiator gene of *Vibrio cholerae* chromosome II. J Bacteriol *187*, 7167-7175.

Ramachandran, R., Ciaccia, P.N., Filsuf, T.A., Jha, J.K., and Chattoraj, D.K. (2018). Chromosome 1 licenses chromosome 2 replication in *Vibrio cholerae* by doubling the *crtS* gene dosage. PLoS Genet *14*, e1007426.

Val, M.E., Skovgaard, O., Ducos-Galand, M., Bland, M.J., and Mazel, D. (2012). Genome Engineering in *Vibrio cholerae*: A Feasible Approach to Address Biological Issues. PLoS Genet *8*, e1002472.

Venkova-Canova, T., and Chattoraj, D.K. (2011). Transition from a plasmid to a chromosomal mode of replication entails additional regulators. Proc Natl Acad Sci U S A *108*, 6199-6204.

Venkova-Canova, T., Srivastava, P., and Chattoraj, D.K. (2006). Transcriptional inactivation of a regulatory site for replication of *Vibrio cholerae* chromosome II. Proc Natl Acad Sci U S A *103*, 12051-12056.
